# Explainable machine learning for predicting longitudinal dementia status: Establishing a leakage-free benchmark

**DOI:** 10.1371/journal.pdig.0001409

**Published:** 2026-05-18

**Authors:** Mohammad Mahdi Ghiasi, Ryan Stanley Falck, Teresa Liu-Ambrose, Roger C. Tam

**Affiliations:** 1 School of Biomedical Engineering, University of British Columbia, Vancouver, British Columbia, Canada; 2 Djavad Mowafaghian Centre for Brain Health, University of British Columbia, Vancouver, British Columbia, Canada; 3 Centre for Aging SMART at Vancouver Coastal Health, Vancouver Coastal Health Research Institute, Vancouver, British Columbia, Canada; 4 Department of Physical Therapy, University of British Columbia, Vancouver, British Columbia, Canada; Oxford University: University of Oxford, UNITED KINGDOM OF GREAT BRITAIN AND NORTHERN IRELAND

## Abstract

Dementia research often suffers from methodological pitfalls such as label-information and subject-information leakages. Leveraging the longitudinal OASIS-2 cohort, this study identifies and addresses three critical gaps in prior research: (i) inclusion of Clinical Dementia Rating (CDR) in predictor sets, risking target leakage and inflated performance estimates; (ii) inappropriate splitting strategies for longitudinal data, risking information leakage; and (iii) inadequate incorporation of temporal dynamics in classification models. To address these, CDR was excluded from input features to ensure that model performance reflects genuine predictive signals from demographic and neuroimaging data. A group-aware data-splitting strategy was implemented to maintain longitudinal data integrity and prevent leakage across training and test sets. Several engineered temporal features were also introduced to evaluate their impact on classification performance. To develop classifiers, a kernel-based classifier (support vector classifier, SVC), boosting ensembles (CatBoost, LightGBM), and bagging ensembles (Random Forest, Extra Trees), were employed. Furthermore, Explainable AI (XAI) techniques, including permutation importance and SHapley Additive exPlanations (SHAP), were utilized to interpret predictions of the best models and identify feature contributions. SVC and LightGBM (LGBM), trained on a combined feature set of original and engineered features, outperformed others. SVC achieved higher precision (73.3%) and discriminative power (ROC AUC 89.1%), while LightGBM provided better recall (69.7%) and accuracy (69.7%). XAI analyses revealed that (i) Mini-Mental State Examination and atlas-scaling-factor dynamics dominated SVC decisions, while (ii) education, age, and estimated total intracranial volume were critical for LGBM predictions. A sex-based analysis showed that both SVC and LGBM models performed better among females, which may indicate potential biases in the models. Collectively, this study establishes a transparent benchmark for leakage-free dementia status classification, demonstrating that realistic predictive performance is markedly lower once leakage is eliminated. This benchmark, together with interpretable analyses, provides a reference point for developing more robust longitudinal dementia models.

## Author summary

In our research, we sought to enhance the reliability of machine learning methods for predicting dementia by addressing common flaws in earlier studies. Dementia affects millions worldwide, and accurate early prediction using brain imaging and personal details collected over time could improve diagnosis and care. However, past work often inflated results due to unintended data leaks, such as including direct indicators of dementia in the prediction process or mishandling repeated patient measurements. We overcame these challenges by excluding the leaking information, ensuring no patient data overlapped between training and testing sets, and creating new measures to track changes in brain features and demographics across visits. Testing various models, we identified two that excelled. Our explanations of the models’ decisions highlighted key influences like education level, age, and brain volume shifts. We also noted better performance for women, suggesting areas for reducing bias. Overall, this study offers a transparent foundation for developing fairer, more effective tools to support dementia research and clinical practice.

## Introduction

### An overview

Dementia is a progressive neurodegenerative disorder that leads to a decline in cognitive function and the ability to perform daily activities [1,2]. It includes various subtypes, with Alzheimer’s disease being the most common [3–5]. One of the most widely used clinical measures in dementia research is the Clinical Dementia Rating (CDR) scale, an ordinal scale that ranges from 0 (no dementia) to 3 (severe dementia), with intermediate values of 0.5, 1, and 2 representing very mild, mild, and moderate dementia, respectively [6–8]. Artificial intelligence (AI), particularly machine learning (ML), has emerged as a powerful tool for analyzing large-scale clinical and neuroimaging datasets, offering the potential to enhance dementia diagnosis and care by identifying patterns that may be difficult to detect [9–13].

Longitudinal studies play a critical role in understanding dementia progression, as they capture temporal changes in cognitive and neuroimaging markers, enabling the development of predictive models for early diagnosis and disease monitoring. For instance, the study by Nguyen et al. [14] introduces a deep recurrent neural network (RNN) model, specifically a minimalRNN, to predict the longitudinal progression of Alzheimer’s disease using data from the Alzheimer’s Disease Neuroimaging Initiative (ADNI). The model was trained to predict future clinical and biomarker observations, such as CDR scale and brain volumes respectively, using a sequence of prior observations, with a focus on handling missing data through strategies like forward filling, linear filling, and a novel model filling approach. The RNN was compared against baselines with the minimalRNN demonstrating superior performance in forecasting Alzheimer’s progression. Similarly, Bhagwat et al. [15] employed ADNI and the Australian Imaging, Biomarker and Lifestyle (AIBL) cohorts to model and predict Alzheimer’s disease symptom trajectories, using hierarchical clustering for stable and declining trajectories and a Longitudinal Siamese Network for prediction with multimodal longitudinal data, achieving high accuracy and offering clinical utility for early prognosis. In another study, Huang et al. [16] utilized longitudinal ADNI data to predict Alzheimer’s disease conversion, employing a voxel-based hierarchical classification framework that leverages longitudinal changes and spatial contiguity. They achieved 79.4% accuracy and 86.5% sensitivity, outperforming baseline and single-classifier methods.

One of the available public datasets that have been employed in the literature is the Open Access Series of Imaging Studies (OASIS)-2 dataset [17]. This dataset includes longitudinal MRI scans, clinical assessments and demographic information, and provides a valuable resource for ML-based classification of dementia. It enables the distinction between patients with dementia, patients without dementia, and converted patients (individuals initially diagnosed without dementia who later developed dementia), facilitating the development of models that may capture disease progression and support early diagnosis. Despite its widespread use, different studies have taken varied methodological approaches in leveraging ML models for dementia classification, leading to diverse findings regarding model performance and classification effectiveness. Next section reviews key ML-based studies utilizing OASIS-2, highlighting their contributions and limitations. Then, the identified gaps in these studies motivate this research, which is discussed, along with the specific objectives of this work.

### ML models for OASIS-2: a review of existing literature

Using the OASIS-2 dataset, Waldo-Benítez et al. [18] employed Support Vector Machine (SVM), Naïve Bayes (NB), Decision Tree (DT), Random Forest (RF) and k-Nearest Neighbors (k-NN) for classification modeling. Seven input features were selected based on a correlation map: sex, age, education level (EDUC), Mini-Mental State Examination (MMSE) scores, CDR, normalized whole brain volume (nWBV) and atlas scaling factor (ASF). The target for prediction is the classification of patients into three groups, including with dementia, without dementia, and converted, based on the selected features. The k-NN algorithm achieved the highest accuracy at 92.1% ± 3.5, followed by RF with 92.0% ± 1.8. The inclusion of CDR in the input set may risk target leakage, as CDR directly reflects dementia severity (or its absence), potentially inflating model performance by providing diagnostic information during training.

Arora et al. [19] developed multi-class ML models for classifying the OASIS-2 participants into with dementia, without dementia and converted using Logistic Regression (LR), Extra Trees (ET), RF, XGBoost (XGB), LightGBM (LGBM), DT, Gradient Boosting, Gaussian NB and SVM. Gradient Boosting achieved the highest accuracy (96.0%), along with the best recall (90.8%) and F1-score (92.0%), while XGB outperformed other models in precision (96.5%). The paper lists all fifteen features of the OASIS-2 dataset, including CDR. However, it states that a correlation matrix was used to select a subset of these features for building the ML models, but the paper does not specify which features were included in the selected features for modeling. Therefore, the lack of a clear list of final features after correlation matrix analysis makes it uncertain whether CDR was included in the modeling process.

Battineni et al. [20] employed the SVM method for OASIS-2 classification. The authors experimented with different kernel functions, gamma values, and regularization parameters to optimize the classification performance. Age, MMSE, CDR, MR Delay, and nWBV were selected as input features to classify subjects into without dementia, with dementia, and converted groups. The optimal hyperplane used low RBF (1.0E-4) and high C (100), yielding 150 support vectors with a bias of 0.3. The model achieved a performance of 70% overall accuracy. The study does not mention train-test split and feature standardization, which are critical for SVM performance.

Sivakani and Ansari [21] performed feature extraction using the Expectation-Maximization algorithm, clustering the data into five groups, followed by feature selection using the Best First algorithm. However, the specific selected features were not provided in the study. Classification was conducted with Gaussian Process, Linear Regression, and Decision Stump algorithms. The Gaussian Process model demonstrated the best performance, evaluated through metrics such as correlation coefficient, mean absolute error, root mean squared error, relative absolute error, and root relative squared error. However, the classification target was not explicitly defined by the authors, making the interpretations complicated. Furthermore, the authors did not report standard classification metrics, including accuracy, precision, and recall.

Basheer et al. [22] introduced a modified Capsule Network (MCapNet) for dementia prediction, focusing on hierarchical feature extraction and optimization techniques. Unlike conventional deep learning models such as Convolutional Neural Networks (CNNs), which rely on large-scale datasets, CapNets retain spatial hierarchies and relationships between features. The proposed model integrated exploratory data analysis (EDA) to assess feature importance, followed by feature selection and optimization to improve computational efficiency. The study applied principal component analysis (PCA) for dimensionality reduction and utilized autoencoders to extract meaningful representations from the data. The MCapNet model achieved a classification accuracy, recall and F-Score of 92.3%, 82.3% and 88.8%, respectively. The study further conducted an ablation study, comparing the MCapNet model with other ML frameworks, like DTs, ensembles and Support Vector Classifier (SVC). Results indicated that the hierarchical structure of CapNets provided a more robust feature representation, leading to improved classification performance. However, the study lacks details about which features are selected or excluded beyond the ablation study.

Baglat et al. [23] applied LR, SVM, DT, RF, and adaptive boosting for binary classification of the OASIS-2 dataset (with dementia vs. without dementia). The highest accuracy (86%) was achieved using RF and the adaptive boosting classifier. The lack of transparency and specificity regarding the input feature set hinders a thorough evaluation of this study. The study did not provide an explicit list of all input features used for the ML models, instead introducing only the general structure of the OASIS-2 dataset. This omission creates uncertainty about whether critical features, such as CDR, were included as inputs.

Kavitha et al. [24] evaluated multiple classifiers, including DT, RF, SVM, and XGB, using the OASIS-2 dataset. Correlation coefficient, Information gain, and Chi-square methods were applied as feature selection methods. The best-performing models were RF and XGB. RF achieved an accuracy of 86.9%, with a precision of 85.0%, recall of 81.0%, and an F1-score of 80.0%, while XGB attained an accuracy of 85.9%, precision of 85.0%, recall of 83.0%, and an F1-score of 85.0%. The confusion-matrix dimensions and class counts indicate a two-class problem that maps *CDR > 0* to with dementia and *CDR = 0* to without dementia. The handling of the converted group is unclear. They are likely merged into with dementia class for visits where CDR ≥ 0.5, as the paper does not mention a multi-class approach or excluding converted patients. Because the label is defined from CDR, passing the same variable into the model introduces target leakage and inflates performance. No experiment removes CDR to quantify genuine biomarker signal.

Pettersson [25] conducted a binary classification study using the OASIS-2 dataset. Three dataset variations were created: unmoderated dataset (containing missing values), no missing values dataset (samples with missing values removed), and reduced attributes dataset (removing low-importance attributes). The author applied DT algorithms to each variant derived from OASIS-2. For OASIS-2, the Classification and Regression Tree (CART) algorithm achieved the highest accuracy of 82.5% when missing values were removed. For the reduced attributes OASIS-2 dataset, a correlation-based feature selection subset evaluator was applied and sex, nWBV, MMSE and CDR were retained. On the other hand, the CDR’s value was used to classify the data into Alzheimer’s disease category if CDR > 0 or normal category if CDR = 0. Thus, CDR appears simultaneously as an input feature and as the rule that generates the class label, creating pronounced label-information leakage. The reported 82.5% accuracy therefore reflects the model’s access to a near-deterministic predictor rather than its ability to learn independent imaging or demographic biomarkers, limiting the generalisability of the findings.

Recently, Dhakal et al. [26] developed a ML framework for a classification of OASIS-2 data by applying nine supervised learning algorithms. The target variable is described as having two values, including with dementia and without dementia. The paper does not explicitly state how the converted group was handled. However, given the binary classification, it is likely that the converted group was either merged with dementia group or excluded. The paper employs Least Absolute Shrinkage and Selection Operator (LASSO) and Chi-square feature selection methods. For the LASSO approach, the final selected features are not listed. On the other hand, the Chi-square method selected CDR, MMSE, eTIV, sex, EDUC, and SES as input features. The highest accuracy (96.77%) was achieved using SVM with the full set of features, while k-NN yielded the best F1-score (94.54%) and recall (100%) when using LASSO-selected features. However, Given CDR’s role in defining the Group label in OASIS-2, its inclusion as an input feature raises concerns about target leakage.

More recently, Alshamlan et al. [27] trained SVM, RF, and LR classifiers on the OASIS-2 dataset. The paper notes that feature selection was performed using three methodologies: Minimum Redundancy Maximum Relevance (mRMR), Correlation Coefficient, and Mutual Information (MI), selecting a subset of five features for modeling. LR with mRMR achieved the highest accuracy (99.08%), while SVM and RF also performed well, with accuracies ranging from 91.43% to 98.16%, depending on the feature selection method used. All the applied feature selection methods consistently ranked CDR among the top five predictors. Furthermore, models were trained using all features, excluding target, to compare performance. Although the OASIS-2 dataset has three group labels, including with dementia, without dementia and converted, the authors combined the converted category with the dementia category to create a binary classification.

Ribino et al. [28] utilized unsupervised ML techniques to perform an analysis of the OASIS-2 longitudinal dementia dataset. This study did not predict a specific target variable but aimed to identify stable patient clusters across the first three visits, with visit 4 included but noted for its small sample size of 14 patients. Additionally, patients with missing variables were excluded, reducing the final sample size to 142 individuals. For their unsupervised ML approach, the authors employed eight input features for clustering, including sex, age, EDUC, SES, MMSE, eTIV, nWBV, and ASF. To maintain an unsupervised approach, CDR was omitted from the input features. Instead, CDR is used solely as a validation target to interpret the clinical significance of the clusters after they are formed. Several techniques, including k-means, Hierarchical Clustering, Gaussian Mixture Model, and Spectral Clustering, were applied. Clustering results showed that Hierarchical Clustering performed best overall, achieving 61.6% accuracy for healthy patients and 64.3% for cognitively impaired individuals. The Gaussian Mixture Model and Spectral Clustering were more effective at grouping healthy patients (69.8% accuracy) but struggled with cognitively impaired individuals (46.4% and 35.7%, respectively). The hierarchical model demonstrated balanced performance in classifying both groups, making it the most reliable approach in this study.

### Knowledge gaps and objectives

Despite the increasing application of a variety of ML algorithms in dementia classification utilizing the OASIS-2 dataset, several methodological challenges persist in existing literature. Addressing these limitations is essential for improving the reliability and generalizability of ML models in dementia research. This study identifies three key gaps in the literature and proposes methodological advancements to enhance dementia classification based on the OASIS-2 dataset.

#### Inclusion of CDR in the input features.

A substantial body of OASIS-2 ML studies report impressive classification performance by including CDR in the predictor set. Representative examples list CDR alongside demographic and MRI-based variables in their feature tables or explicitly pass it through feature-selection pipelines. As the OASIS-2 *Group* label (without dementia, converted, with dementia) is itself derived from CDR, feeding CDR back into the model introduces label-information leakage. This can inflate cross-validation performance and mask the true value of imaging or demographic biomarkers. A work on medical-AI reproducibility by Wen et al. [29] indicated that more than half of published dementia-classification papers still suffer from some form of data leakage, calling for more rigorous experimental designs. However, to the best of the authors’ knowledge, no published OASIS-2 supervised ML study has systematically removed CDR from the input space while retaining clinically available variables (demographics, MRI-derived features) and the readily collected MMSE score. Consequently, the field lacks a leakage-free baseline against which CDR-dependent models can be fairly compared and an assessment of how much predictive signal remains once CDR is excluded.

#### Limitations in data splitting strategies.

A significant limitation in prior work is the handling of longitudinal data within the OASIS-2 dataset. The reviewed AI/ML modeling studies utilizing the OASIS-2 dataset did not indicate that they accounted for the longitudinal nature of the data in designing their splitting strategies. Since OASIS-2 contains repeated measures for each subject, observations are clustered within subjects rather than being independent. Models trained without accounting for this could unintentionally memorize specific subjects, particularly if their data appears in both the training and test sets, leading to overly optimistic performance estimates and hindering generalization to unseen subjects. Therefore, the use of standard random data-splitting techniques may introduce data leakage, wherein information from the same subject appears in both training and test sets. To address this issue, this study employs a group-aware data-splitting technique to ensure that all data from a given subject is confined to either the training or test set. This approach preserves the integrity of the evaluation process, ensuring the model’s performance accurately reflects its ability to generalize beyond familiar subjects. This strategy not only enhances prediction validity but also establishes a more robust framework for evaluating model performance on the OASIS-2 dataset, preventing performance inflation caused by data leakage and addressing a critical gap in existing AI/ML studies.

#### Limited integration of temporal dynamics.

Given that dementia is a progressive condition, analyzing temporal variations in clinical and neuroimaging markers is important for early-stage detection and disease progression modeling. However, existing studies have primarily relied on feature selection and extraction methods to enhance model performance, which often operate on static data representations that do not account for longitudinal changes. Feature selection methods, such as LASSO [30,31] and Chi-square tests [32,33], identify and retain the most important features from a dataset. However, these features are typically selected independently of time, meaning they do not capture how values change across different visits. Similarly, feature extraction methods, such as PCA [34,35], transform raw data into lower-dimensional feature sets while preserving essential information. Although effective in reducing dimensionality, these techniques are generally applied without considering temporal dependencies. To address this limitation, this study introduces new features that capture temporal changes in clinical and neuroimaging data, an aspect largely unexplored in previous research utilizing OASIS-2. The longitudinal structure of the data offers a unique opportunity to analyze how these features evolve over time, providing deeper insights into disease progression. By capturing these temporal dynamics, the study aims to enhance the predictive power of the models, offering a more nuanced understanding of dementia onset and progression.

#### Research objectives.

In response to the identified gaps, the primary objective of this study is not merely to improve predictive performance, but to establish a rigorous, leakage-free benchmark for dementia classification using the OASIS-2 dataset. Specifically, this study aims to: (1) implement a group-aware data-splitting strategy to strictly isolate subjects and prevent data leakage; (2) quantify the realistic predictive power of standard ML models when CDR is removed from the input set; and (3) evaluate whether novel engineered temporal features can recover meaningful predictive signals in this strictly constrained environment. A range of models including SVC [36,37], boosting ensembles such as CatBoost (CB) [38], and LGBM [39], as well as bagging ensembles like RF [40,41] and ET [42,43] are employed.

Additionally, Explainable AI (XAI) techniques, such as permutation importance [44] and SHapley Additive exPlanations (SHAP) [45], are employed to interpret model predictions, revealing the contributions of time-based neuroimaging and demographic features to dementia classification. By systematically evaluating these models using statistical performance metrics, the most suitable approach for dementia classification within the context of the OASIS-2 dataset can be determined. XAI methods further provide interpretable insights into key predictors, supporting clinical applicability. Additionally, this study seeks to derive new insights from the dataset through EDA.

To achieve these objectives, the rest of the paper is structured as follows: Section 2 provides an overview of the OASIS-2 dataset, including feature distribution analysis and trend analysis. Section 3 details the methodology, covering encoding techniques, feature engineering, handling class imbalance, and feature scaling. Section 4 presents the results and discussion, comparing model performance, analyzing key findings, and analyzing XAI-derived feature contributions. Finally, Section 5 concludes the study, summarizing its contributions and highlighting potential future research directions.

## Materials and methods

### Overview of the OASIS-2 dataset

The OASIS-2 dataset [17] comprises a longitudinal cohort of 150 right-handed individuals aged between 60 and 96 years. It consists of male and female subjects. Each participant underwent multiple imaging sessions, with a total of 373 magnetic resonance imaging (MRI) sessions included in the dataset. These sessions provide 3 or 4 T1-weighted MRI scans per subject. At the initial scan, 72 individuals were classified as without dementia (190 imaging sessions) and remained so throughout the study. Meanwhile, 64 subjects were diagnosed with dementia at their first visit and exhibited sustained dementia in subsequent scans (146 imaging sessions), with 51 of these individuals having mild to moderate Alzheimer’s disease. Additionally, 14 participants were initially without dementia but were later diagnosed with dementia during the course of the study (37 imaging sessions). The OASIS-2 dataset includes a variety of features including Subject ID, MRI ID, group (categorizing subjects as with dementia, without dementia or converted), visit (indicating the time point of the scan), MR delay (time since the previous scan in days), sex (male or female), age, socioeconomic status (SES), EDUC, MMSE scores, CDR, estimated total intracranial volume (eTIV), nWBV and ASF. The MRI ID column was removed from the dataset as it was a unique identifier that did not contribute to the classification analysis. Detailed information about each feature is summarized in Table 1. These features, along with the longitudinal structure of the dataset, provide a comprehensive framework for investigating brain volume changes and cognitive decline in aging and dementia.

**Table 1:** Descriptive statistics of demographic, cognitive, and imaging features for without dementia, with dementia, and converted groups in the OASIS-2 dataset.

Without dementia (32.1% Male and 67.9% Female)							
Feature	mean	std	min	25%	50%	75%	max
Visit	1.97	0.98	1	1	2	2.75	5
MR Delay (Days)	739	243	182	582	704	846	1510
Age (Years)	77.06	8.10	60	71	77	82	97
EDUC (Years)	15.14	2.74	8	13	16	18	23
SES	2.39	1.05	1	2	2	3	5
MMSE	29.23	0.88	26	29	29	30	30
CDR	0.01	0.05	0	0	0	0	0.5
eTIV (cm^3^)	1495.47	184.93	1105.65	1358.05	1474.51	1634.69	2004.48
nWBV	0.74	0.04	0.64	0.72	0.74	0.77	0.84
ASF	1.19	0.14	0.88	1.07	1.19	1.29	1.59
**With dementia (58.9% Male and 41.1% Female)**							
Feature	**mean**	**std**	**min**	**25%**	**50%**	**75%**	**max**
Visit	1.73	0.79	1	1	2	2	5
MR Delay (Days)	651	229	212	520	590	758	1455
Age (Years)	76.26	6.94	61	71	76	81	98
EDUC (Years)	13.67	2.9	6	12	13	16	20
SES	2.77	1.2	1	2	3	4	5
MMSE	24.51	4.5	4	22	26	28	30
CDR	0.67	0.3	0.5	0.5	0.5	1	2
eTIV (cm^3^)	1485.85	173.72	1143.11	1356.99	1476.46	1566.84	1956.96
nWBV	0.72	0.03	0.65	0.69	0.71	0.74	0.81
ASF	1.2	0.14	0.9	1.12	1.19	1.29	1.54
**Converted (35.1% Male and 64.9% Female)**							
Feature	**mean**	**std**	**min**	**25%**	**50%**	**75%**	**max**
Visit	2.03	1.04	1	1	2	3	5
MR Delay (Days)	858	387	191	627	774	1059	1707
Age (Years)	79.76	7.43	65	74	81	86	92
EDUC (Years)	15.46	2.52	12	14	16	18	20
SES	1.73	0.96	1	1	1	2	4
MMSE	28.68	1.56	24	28	29	30	30
CDR	0.26	0.25	0	0	0.5	0.5	0.5
eTIV (cm^3^)	1459.35	135.38	1264.40	1383.37	1422.62	1586.52	1721.81
nWBV	0.72	0.04	0.67	0.70	0.72	0.75	0.80
ASF	1.21	0.11	1.02	1.11	1.23	1.27	1.39

### Feature distributions

**Fig 1:**
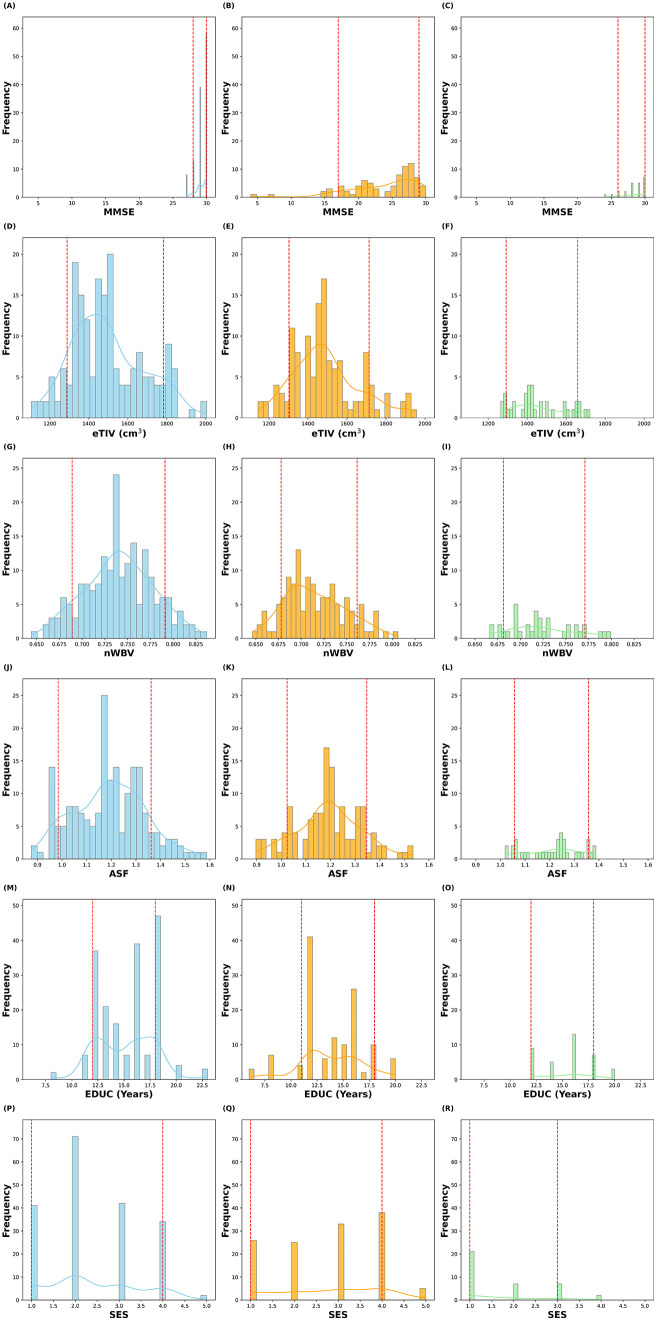
Distributions of various features for each group in the OASIS-2 Dataset. The without dementia, with dementia and converted groups are shown by blue, orange and green, respectively. The red dashed lines indicate the 10^th^ and 90^th^ percentiles for each group, while the smooth curves represent kernel density estimations for each group’s distribution.

The feature distributions for the OASIS-2 dataset were analyzed across three distinct groups, including without dementia, with dementia, and converted individuals. Distributions of six key features, MMSE, eTIV, nWBV, ASF, EDUC and SES, were plotted, and the results are shown in Fig 1. Feature distributions were visualized using histograms overlaid with kernel density estimation curves to illustrate the spread and central tendency for each group. The 10^th^ and 90^th^ percentiles were marked to indicate the range of variation in feature values across groups. These plots provide insight into how each feature varies across the three groups.

S1 Appendix provides a comprehensive trend analysis of CDR within the OASIS-2 dataset, exploring its relationship with key neuroimaging, demographic, and clinical variables. The analysis aims to demonstrate how factors such as age, MMSE scores, nWBV, eTIV, and ASF change as subjects transition from CDR = 0 to CDR > 0 or progress to more severe cognitive impairment stages.

### Encoding

As the data was already checked for missing values (see S1 Appendix for details), the next step in preparing the data for dementia status classification involved encoding categorical variables. Proper encoding is crucial, as certain algorithms, such as LR or distance-based methods like k-NN, may incorrectly interpret numerical encodings as having an ordinal relationship, potentially biasing model performance. For the ‘M/F’ (sex) column, using label encoding (e.g., ‘M’ = 0, ‘F’ = 1) would introduce an implicit order (‘M’ < ‘F’), which could mislead algorithms that interpret numerical values ordinally. Instead, One-Hot Encoding was applied to create separate binary columns for each category, ensuring no artificial ordinal relationships were introduced. Although ‘M/F’ is binary, One-Hot Encoding prevents potential misinterpretations across all algorithms, particularly those sensitive to numerical scales or order. This encoding resulted in a binary column ‘M/F_M’, where 1 represents male and 0 represents female. In the case of the ‘Group’ column, as it is the target feature in the classification, tree-based methods treat the target as categorical, irrespective of its numerical encoding. Therefore, label encoding can still be applied to this column. However, for algorithms sensitive to feature scales and orders, One-Hot encoding should be utilized to prevent the model from assuming any ordinal relationship among ‘with dementia,’ ‘without dementia’ and ‘converted.’

### Feature engineering

The dataset under consideration comprises longitudinal clinical and imaging data collected from subjects over multiple visits. Each subject has undergone two to five scans, resulting in repeated measurements that capture temporal changes in various clinical and neuroimaging features. The goal of this feature engineering process is to transform and enhance the data to improve the predictive power of ML models aimed at classifying dementia status. Prior to feature engineering, unnecessary columns such as ‘Hand’ and ‘MRI ID’ were removed due to their irrelevance to the predictive task. Given the longitudinal nature of the data, it is essential to account for the temporal sequence of observations. Incorporating temporal changes in these variables can enhance the model’s ability to capture trends and patterns associated with disease progression or cognitive decline. Thus, the data was sorted by ‘Subject ID’ and ‘Visit’ to ensure that chronological order was maintained. This ordering is critical for accurately calculating time-based features and capturing the progression of clinical and imaging measures over time.

The data represents repeated measures for each subject. This means that observations (rows) are not independent but are clustered within subjects. While ‘Subject ID’ is an identifier, including it directly as a feature could inadvertently allow the model to memorize subjects rather than learn generalizable patterns. Therefore, if data from the same subject appears in both the training and test sets, it can lead to overly optimistic performance estimates due to the model’s familiarity with the subject. To handle this, the ‘Subject ID’ was excluded from the feature set to avoid the model overfitting to specific subjects.

In this study, feature engineering focused on creating new variables that can capture temporal dynamics and subject-specific changes. Importantly, all engineered temporal features were derived exclusively from within-subject information, using only past or concurrent observations for each individual visit. No information from future visits or from other subjects was used in the construction of any feature, ensuring that the feature engineering process itself does not introduce temporal or subject-level information leakage.

The following features are engineered to enhance the model’s ability to learn from the longitudinal data. For each subject, the age at their first visit was recorded and served as a reference point for subsequent age-related calculations. Another age-related feature included the difference between the age at each visit and the baseline age. This feature captures the progression of age over the course of the study for each subject. Additionally, the initial MMSE score, initial CDR score, initial nWBV measurement and initial SES were stored for each subject. Other engineered features included the change in MMSE, CDR, nWBV, eTIV, ASF, EDUC and SES between consecutive visits which were computed using the difference between the current score and that from the previous visit for each subject. Eq. (1) shows the formula.


$$\begin{array}{c} x_{change}=x_{current}-x_{previous} \end{array}$$ (1)

where *x* represents the feature of interest. As there were no changes in the EDUC and SES values between consecutive visits, the newly created features from these columns were removed from the inputs before proceeding with modeling.

Furthermore, the cumulative changes in MMSE, CDR, nWBV, eTIV and ASF were calculated to capture the overall change in cognitive function since the beginning of the study, the overall progression of dementia severity, long-term changes in brain volume, potential structural brain changes associated with neurodegeneration and possible alterations in brain size relative to a standard template over time, respectively. Eq. (2) shows the formula.


$$\begin{array}{c} x_{cumulative\;change}=x_{current}-x_{baseline} \end{array}$$ (2)

The rate of change for MMSE, nWBV, eTIV and ASF are other created features that helped capture the speed of cognitive change or structural atrophy over time. Eq. (3) shows the formula.


$$\begin{array}{c} x_{rate\;of\;change}=\frac{x_{cumulative\;change}}{\;days\;since\;baseline} \end{array}$$ (3)

Additionally, a feature called visit interval was introduced, representing the number of days between consecutive visits for each subject. This feature captures irregular time gaps between assessments, which may influence the rate of change in clinical measures and impact the modeling of disease progression.

### Handling class imbalance

Class imbalance is a critical challenge in ML classification tasks, particularly in datasets where certain classes are underrepresented. In the OASIS-2 dataset, the class distribution consists of Class 0 (Converted) with 37 samples, Class 1 (with dementia) with 146 samples, and Class 2 (without dementia) with 190 samples. The significant disparity between these class sizes can lead to biased models that favour the majority classes while underperforming in detecting minority class instances. Addressing this imbalance is essential to ensure that the model generalizes effectively and provides reliable predictions across all dementia categories. To mitigate class imbalance, the Synthetic Minority Over-sampling Technique (SMOTE) with Tomek Links [46–48] was applied to the training data. SMOTE generates synthetic samples for the minority class by interpolating feature values between existing observations, while Tomek Links helps refine the dataset by removing borderline or overlapping samples, reducing noise and improving class separability.

Since standard SMOTE does not inherently account for group constraints, specific precautions were taken to prevent data leakage and maintain model validity. First, the dataset was split in a way that ensured entire subject groups were assigned exclusively to either the training or testing set. This approach eliminated subject overlap, reducing the risk of the model memorizing individual subjects instead of learning generalizable patterns. Next, SMOTE was applied solely to the training set, ensuring that synthetic samples were generated within the training data without introducing any information from the test set. This step preserved the integrity of the evaluation process, preventing information leakage between training and testing sets. While grouping during data splitting helps minimize leakage risks, applying SMOTE without explicit group constraints may still introduce biases if the synthetic samples capture interdependencies within subject groups.

### Model development

The model development phase began by first sorting the OASIS-2 dataset by subject identifier and visit number to maintain the longitudinal sequence of observations for each individual. To preserve subject independence, a single group-aware train–test partition was created with 80% of subjects allocated to the training set and the remaining 20% to the test set. This procedure guaranteed that an entire individual’s records were confined to either training or testing. Following the split, feature scaling was applied to both training and test sets, thereby ensuring consistent feature distributions and mitigating scale discrepancies.

A random hyperparameter search was then conducted for the SVC, ET, RF, CB, and LGBM models wrapped in a calibration procedure for probability estimates. This search involved randomly sampling values from predefined hyperparameter ranges for each model. During this phase, hyperparameter configurations were evaluated using group-aware 5-fold cross-validation on the training set alone, where the training set was divided into five folds, ensuring that all records for a subject remained within the same fold. Each fold served as a validation set once, while the remaining four folds formed the training data for that iteration. The held-out test set was strictly reserved for final evaluation and was not used during hyperparameter tuning, ensuring that model performance reflected generalization to unseen data. After identifying the best hyperparameter settings, the study retrained the final models on the entire training set using group-aware 5-fold cross-validation and comprehensively evaluated them on the held-out 20% test set. The confusion matrices reported in the results are derived from this single test set, reflecting the models’ performance on unseen subjects.

**Fig 2:**
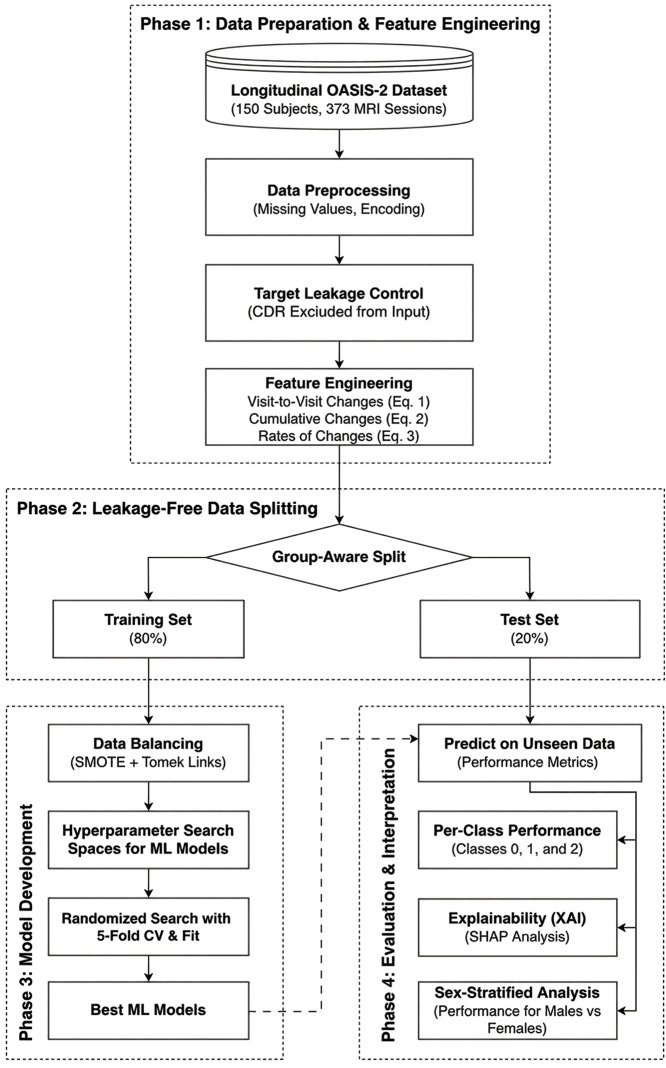
Schematic overview of the leakage-free methodological framework for dementia status classification using the OASIS-2 dataset.

Multiple metrics were recorded, including accuracy, precision, recall, F1-score, receiver operating characteristic (ROC) area under the curve (AUC) and precision-recall (PR) AUC. These metrics provide a comprehensive evaluation, highlighting how well each model distinguishes between diagnostic groups. By retaining one definitive test partition that contained no overlap with the training data, the study obtained a final, unbiased assessment of how well the model would classify new patients in clinical or research applications. This single-pass, group-aware approach struck a balance between methodological rigor, preventing data leakage and overfitting, and practical considerations, such as computational efficiency, while still providing a realistic gauge of model performance on longitudinal dementia data. Fig 2 illustrates the methodological framework, structured into four distinct phases: data preparation and feature engineering, group-aware data splitting, machine learning model training, and evaluation with interpretation.

## Results and discussion

### Comparison of the models

The error analysis results for each developed model on the test dataset are presented in Table 2. These outcomes provide a detailed assessment of the models’ performance specifically on unseen data, highlighting their ability to generalize beyond the training set. Every model was trained and evaluated twice: first with the original OASIS-2 attributes, and then with an expanded feature space that incorporates both the original features and engineered visit-to-visit temporal descriptors. This can facilitate a direct comparison of how longitudinal context reshapes classifier behaviour when distinguishing diagnostic label of participants in OASIS-2.

**Table 2:** Error analysis for the test dataset used to evaluate the developed ML models based on different feature sets.

Model	Input	Metrics (%)					
		Precision	Recall	F1-score	ROC AUC	PR AUC	Accuracy
CB	Original	65.8	64.5	63.9	82.7	66.3	64.5
	Combined^*^	63.9	60.5	59.3	84.0	65.5	60.5
ET	Original	64.1	60.5	59.9	78.7	69.4	60.5
	Combined	66.0	57.9	57.6	81.2	63.7	57.9
RF	Original	69.1	69.7	68.5	80.3	70.4	69.7
	Combined	63.6	68.4	65.6	77.6	62.2	68.4
LGBM	Original	65.7	65.8	64.6	83.9	74.8	65.8
	Combined	72.5	**69.7**	**69.6**	81.2	61.2	**69.7**
SVC	Original	65.4	63.2	63.4	85.3	74.2	63.2
	Combined	**73.3**	68.4	69.0	**89.1**	**76.2**	68.4

* Both original and engineered temporal features

Using the original OASIS-2 variables only, RF yielded the highest threshold-dependent scores, including precision, recall, F1, and accuracy. However, ranking-based criteria diverged: SVC achieved the top ROC AUC, indicating the most reliable global ordering of class probabilities, whereas LGBM attained the highest PR AUC, which reflects the best retrieval of the minority “converted” class. Conversely, ET produced the lowest values for every metric except PR-AUC; the weakest PR AUC, however, was observed with CB, indicating that while CB might perform adequately in other areas, its ability to prioritize and correctly identify positive cases was comparatively less effective than other models with the original feature set. These patterns suggest that RF optimises class assignment, while SVC and LGBM provide a better probability calibration and minority-class detection.

Across the evaluated models, the use of engineered time-based features alongside the original features yielded mixed results. For the RF model, the inclusion of engineered features alongside the original feature set resulted in a consistent decrease across all performance metrics. For the CB model, the combined feature set led to a slight decrease in precision, recall, accuracy, F1-score, and PR AUC, despite a marginal improvement in ROC AUC (from 82.7% to 84.0%). This suggests that while the model might be better at creating more separation between positive cases and negative ones with the added features (higher ROC AUC), its overall ability to correctly classify all instances decreased. Similarly, for ET, the combined feature set resulted in a decrease in most metrics, indicating that the engineered features did not consistently improve performance for this model.

Several factors may explain the limited and model-dependent impact of the engineered temporal features. The longitudinal sampling in OASIS-2 is relatively sparse, with most subjects contributing only two to three visits and with variable inter-visit intervals. Deriving rates of change or cumulative differences from such limited observations can amplify measurement noise, thereby reducing the reliability of the resulting temporal descriptors and potentially obscuring underlying disease trajectories. In addition, although the engineered features were intended to capture longitudinal dynamics, they also increased the dimensionality of the feature space. For models such as RF and ET, this added complexity may have outweighed the potential information gain, resulting in degraded classification performance.

The SVC and LGBM models with the combined feature set emerged as the top-performing models for classifying dementia status in the OASIS-2 dataset. These models exhibit comparable performance, with nuanced differences across their evaluation metrics. SVC slightly outperforms LGBM in precision (73.3% vs. 72.5%), indicating a marginally better ability to minimize false positives, which is important in applications where incorrectly classifying a healthy individual as diseased could lead to unnecessary interventions. However, LGBM shows a slight edge in recall (69.7% vs. 68.4%), suggesting it is better at identifying true positives, which is crucial for ensuring diseased individuals are not missed. The F1-scores are nearly identical (69.6% for LGBM vs. 69.0% for SVC), reflecting a balanced trade-off between precision and recall for both models. Overall accuracy is also close, with LGBM at 69.7% and SVC at 68.4%, indicating similar general performance on the test set. These small differences suggest that both models are competitive, but for early dementia prediction, where high sensitivity is critical to minimize false negatives, LGBM may be preferred over SVC, which achieves higher specificity by minimizing false positives. In terms of discriminative power and robustness, the SVC model demonstrates a clear advantage in ROC AUC (89.1% vs. 81.2%) and PR AUC (76.2% vs. 61.2%), highlighting its superior ability to distinguish between classes across various thresholds. The higher ROC AUC for SVC indicates better overall ranking of positive instances over negative ones, while the significantly higher PR AUC suggests it maintains better precision across recall levels, making it more reliable in scenarios where positive cases are rare.

**Fig 3:**
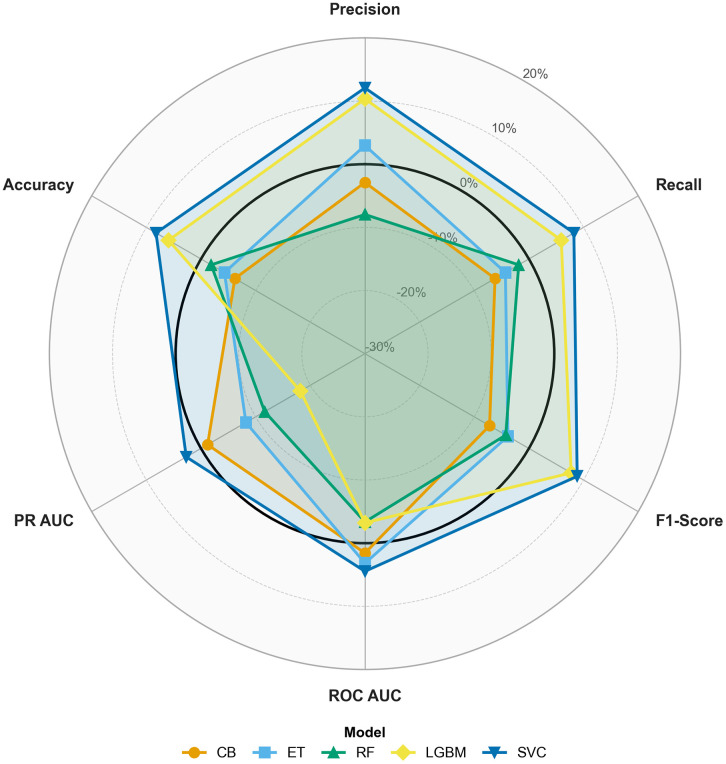
Radar chart showing the percentage change in classification performance across six metrics (Precision, Recall, F1-score, ROC AUC, PR AUC, and Accuracy) when switching from original to combined feature inputs, for each model. The bold black ring denotes 0% change; points beyond it indicate improvement. Percentage change in performance metrics across models after feature engineering: (a) precision, (b) recall, (c) F1-score, (d) ROC AUC, (e) PR AUC and (f) accuracy.

The impact of adding engineered features to the original set of features on model performance is further illustrated in Fig 3. It shows the percentage change in key performance metrics, including precision, recall, F1-score, ROC AUC, PR AUC, and accuracy, across the developed ML models after incorporating engineered features compared to the original feature set. The visualization highlights the varying degrees of changes across models.

To quantify the impact of supplying CDR to the algorithms, the entire modeling pipeline was replicated with CDR added to both feature configurations. In this supplemental experiment each method was re-trained on the original OASIS-2 variables plus CDR, and on the combined set that pairs CDR and created CDR-based features with other engineered features. Performance was then re-evaluated with the identical test protocol used in the main study, enabling a direct, like-for-like comparison with the CDR-excluded models. The complete results of this analysis are presented in S2 Appendix, where the magnitude of performance shifts attributable to CDR can be inspected across all metrics and model families.

Permutation feature importance, as an XAI technique, can be used to evaluate the contribution of individual features to a predictive model’s performance by measuring the impact of randomly shuffling a feature’s values. The method disrupts the relationship between a feature and the target variable by permuting its values while keeping all other features unchanged. The resulting decrease in model performance reflects the feature’s importance. A significant performance drop indicates high feature importance, whereas a minimal change suggests lower significance [49,50]. This approach is advantageous for its applicability across diverse model types, ability to capture feature interactions, and computational efficiency. However, it may be sensitive to correlated features.

**Fig 4:**
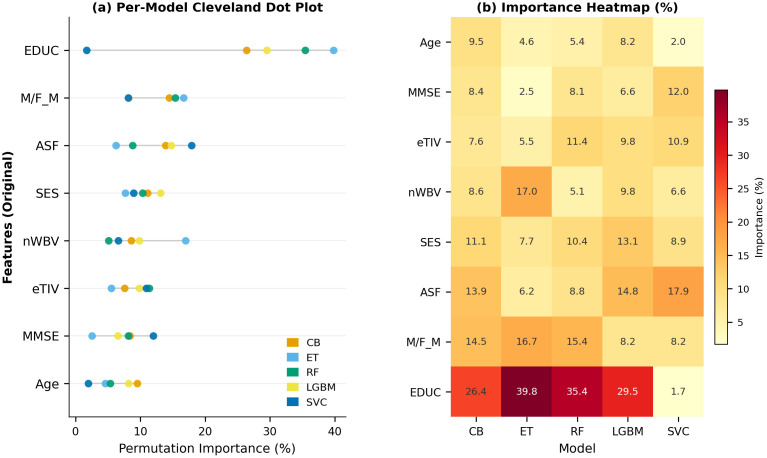
Permutation feature importance across five ML classifiers on the OASIS-2 dataset (original feature set). **(a)** Cleveland dot plot showing per-model importance for each feature, sorted in descending order of mean importance across models. Each dot represents one classifier. **(b)** Heatmap of the same importance values (%), providing a compact numerical summary of the full feature-by-model matrix.

Permutation feature importance for the presented classifiers, trained on the original features, is visualized in Fig 4. As shown, education consistently emerges as the most important feature for all tree-based ensemble models, with importance values ranging from 26.4% (CB) to 39.8% (ET). In contrast, SVC prioritizes ASF (17.89%) with education at a low 1.71%, highlighting model-specific feature sensitivities.

**Fig 5:**
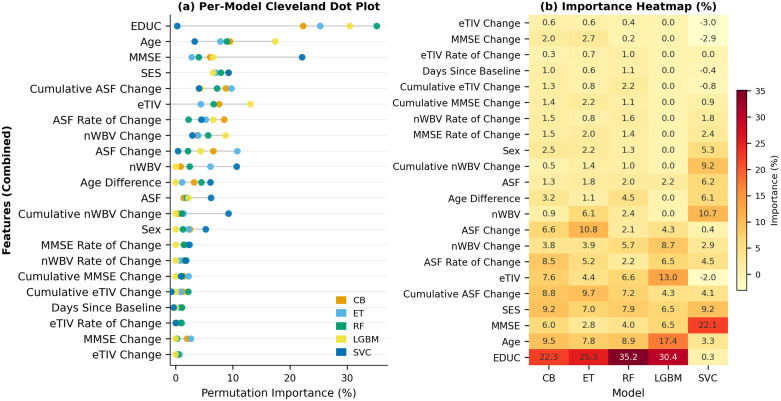
Permutation feature importance across five ML classifiers on the OASIS-2 dataset (combined feature set). **(a)** Cleveland dot plot showing per-model importance for each feature, sorted in descending order of mean importance across models. Each dot represents one classifier. **(b)** Heatmap of the same importance values (%), providing a compact numerical summary of the full feature-by-model matrix.

For the combined feature set, visualized in Fig 5, permutation importance reveals that education remains the dominant feature in tree-based models, with importance values ranging from 22.28% (CB) to 35.16% (RF). In contrast, the SVC model assigns the highest importance to MMSE (22.11%). Notably, some derived features, such as eTIV Change (-3.03%) and MMSE Change (-2.89%) in SVC, exhibit negative importance, indicating that shuffling these features slightly improves performance. This contrast between tree-based and linear models underscores the impact of model architecture on feature prioritization in dementia prediction. The permutation feature importance results for the LGBM model in the combined feature set show several features with zero importance. This indicates that shuffling these features does not affect the LGBM model’s performance, suggesting they contribute negligibly to its predictive power.

### Analysis of the best models

**Fig 6:**
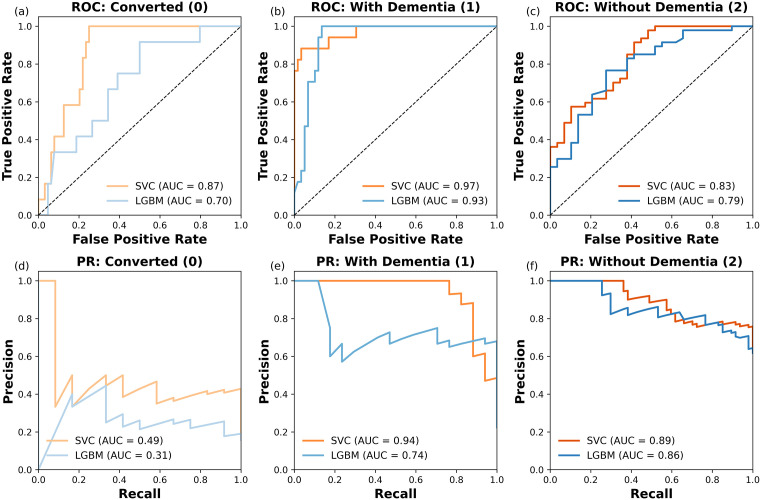
ROC and PR curves for SVC (orange) and LGBM (blue) models across three classes: (a–c) ROC curves for converted (0), with dementia (1), and without dementia (2); (d–f) PR curves for the same classes.

Fig 6 presents ROC and PR curves comparing SVC (orange) and LGBM (blue) models developed based on the combined feature set for classifying converted (Class 0), with dementia (Class 1), and without dementia (Class 2) cases. ROC curves (a–c) show the trade-off between true positive rate and false positive rate, with the ROC AUC indicating overall class separation. PR curves (d–f) focus on precision versus recall, with PR AUC highlighting performance on rare classes.

**Fig 7:**
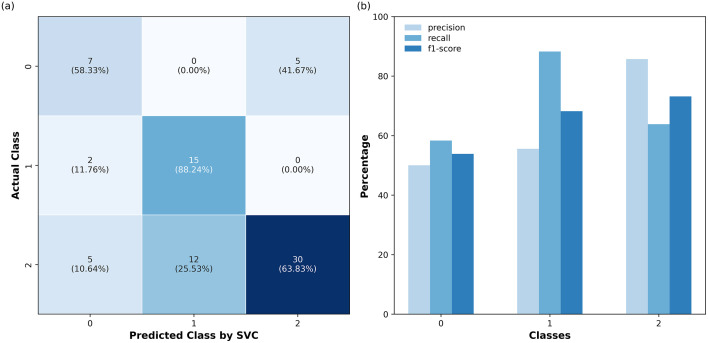
(a) Confusion matrix of the SVC model with combined features displaying both raw counts (top) and percentage values (bottom) for each class. **(b)** Bar chart showing precision, recall, and F1-score for each class in the classification report.

**Fig 8:**
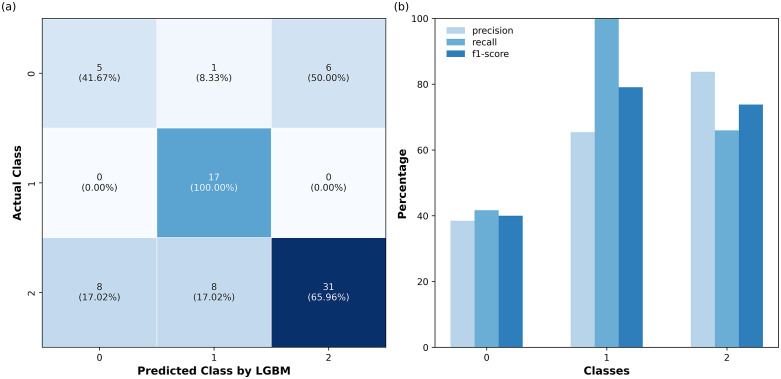
(a) Confusion matrix of the LGBM model with combined features displaying both raw counts (top) and percentage values (bottom) for each class. **(b)** Bar chart showing precision, recall, and F1-score for each class in the classification report.

Figs 7 and 8 show the performance evaluation of the SVC and LGBM models developed based on both original and combined features in predicting the unseen data (test) using two visualizations: a confusion matrix and a bar chart. These combined visualizations offer a comprehensive view of the model’s predictive capabilities, showcasing both the class-specific performance and the overall distribution of predictions. In Figs 7(a) and 8(a), the diagonal entries represent correct classifications, where the model’s predicted labels match the actual labels, while off-diagonal entries denote misclassifications. Figs 7(b) and 8(b) illustrate the precision, recall, and F1-score for each class, providing a detailed breakdown of the model’s accuracy, sensitivity, and overall effectiveness.

The class-specific performance differences provide valuable insights. For Class 0, SVC demonstrates better precision, recall, and F1-score, correctly classifying 7/12 cases compared to LGBM’s 5/12, indicating better identification of this rare class. Despite this relative advantage, both models exhibit limited discriminative ability for Class 0, indicating substantial difficulty in reliably identifying individuals undergoing cognitive conversion. Conversely, for Class 1, LGBM outperforms by classifying all 17 cases correctly, making it highly effective for detecting individuals with dementia, a priority in medical screening. SVC, while slightly less effective, correctly classifies 15/17 with dementia cases, misassigning 2 to Class 0 rather than Class 2. This is less detrimental in a screening context since misclassification as converted still flags the individual for further evaluation, unlike a without dementia label that might dismiss the case. This might make SVC a viable alternative for screening.

However, these class-specific differences should be interpreted with caution given the relatively small size of the held-out test set, particularly for the converted class. With only 12 converted cases available for evaluation, small absolute changes in the number of correct predictions can translate into seemingly large differences in performance metrics. As a result, the observed numerical advantages of SVC for the converted class or LGBM for the dementia class may not be statistically robust. Formal statistical evaluation on larger independent cohorts would be required to determine whether these differences reflect systematic model behavior rather than sampling variability. Furthermore, improving feature engineering or incorporating additional data could help the model better distinguish between classes. More broadly, these findings reinforce that high overall performance metrics are largely driven by the classification of Class 1 and Class 2, while predictive performance for longitudinal conversion remains limited.

### SHAP analysis

To enhance the interpretability of the combined feature set-driven SVC and LGBM models for dementia status classification, SHAP, an XAI technique, was applied to quantify the contributions of original and time-based neuroimaging and demographic features from the OASIS-2 dataset on the test set. This analysis offers complementary perspectives on feature contributions. While permutation importance identifies features critical to predictive accuracy, it assumes feature independence and does not capture interactions. To address this, the SHAP analysis, which assigns instance-level feature contributions while accounting for interactions among features, can be utilized. It elucidates how features drive predictions across three classes by using mean absolute SHAP values.

#### SHAP feature importance findings.

**Fig 9:**
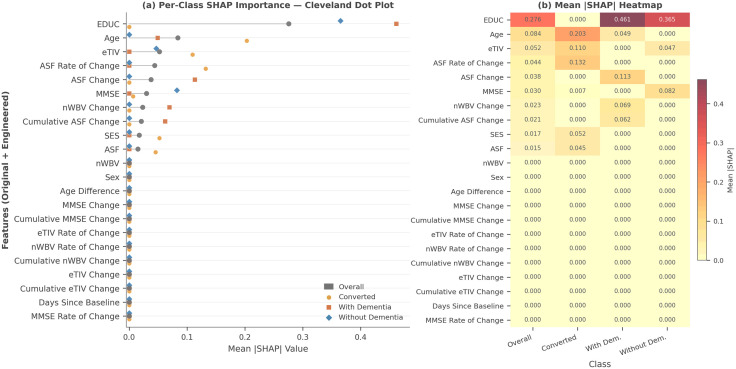
Mean absolute SHAP feature importance for the LGBM model trained with combined features for OASIS-2 classification. **(a)** Grouped Cleveland dot plot showing mean **|**SHAP**|** values per feature for the overall aggregation and each individual class. Features are sorted in descending order of overall mean **|**SHAP**|**. **(b)** Heatmap providing a compact numerical summary of the same mean **|**SHAP**|** values across all features and classes.

The SHAP analysis for the LGBM model revealed distinct feature importance patterns for each class. The SHAP summary plot shown in Fig 9 ranks features by their mean absolute SHAP values for the LGBM model developed based on the combined features. For identifying individuals who converted from without dementia to dementia, the most important features are age, ASF rate of change and eTIV, followed by ASF and SES. MMSE’s contribution is notably minimal for this specific class. On the other hand, EDUC is overwhelmingly the most important feature for classifying individuals with and without dementia. The change in ASF is the next most impactful feature for the with dementia class, followed by nWBV change, cumulative ASF change and age. The second and third most important features for identifying without dementia cases were MMSE and eTIV.

**Fig 10:**
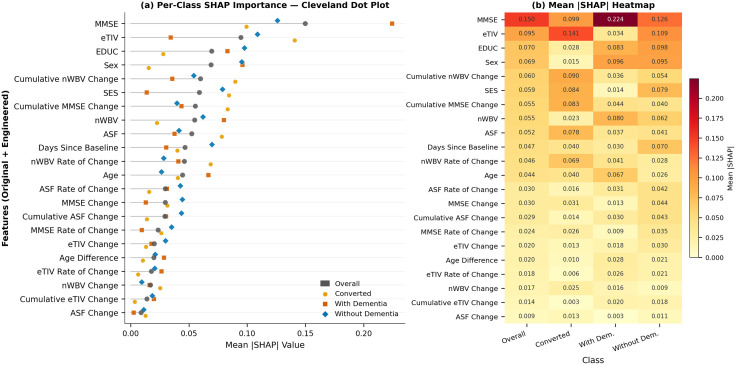
Mean absolute SHAP feature importance for the SVC model trained with combined features for OASIS-2 classification. **(a)** Grouped Cleveland dot plot showing mean **|**SHAP**|** values per feature for the overall aggregation and each individual class. Features are sorted in descending order of overall mean **|**SHAP**|**. **(b)** Heatmap providing a compact numerical summary of the same mean **|**SHAP**|** values across all features and classes.

As can be observed from Fig 10(a), the SVC classifier presents a different feature importance profile, with all features having non-zero SHAP values. MMSE score is the most influential feature for SVC’s overall predictions, emphasizing the importance of cognitive assessment in its decision-making process. eTIV and EDUC are also prominent, similar to LGBM, but their relative ranks differ. Furthermore, sex and cumulative nWBV change are new important features that were less pronounced in the LGBM overall analysis. eTIV plays a significant role in identifying individuals undergoing conversion, i.e., Class 0. MMSE, cumulative nWBV change, cumulative MMSE change, SES, ASF and nWBV rate of change are also highly relevant. However, cumulative eTIV change had near-zero impact. For with dementia class, MMSE is the most impactful feature, which is expected given MMSE’s direct correlation with cognitive impairment. Sex, EDUC, nWBV, and age are the next most important features with similar impact on the model. For without dementia group, although MMSE is the most important feature, its importance is not significantly greater than eTIV, EDUC and sex.

In the case of LGBM, features related to brain volume and demographic variables are prominent, suggesting the model heavily leverages structural brain changes and aging markers to classify conversion trajectories. Furthermore, features show class-specific SHAP values, emphasizing that their importance differs depending on whether the target is converted, with dementia, or without dementia. LGBM may be better at detecting subtle anatomical changes, especially those associated with conversion (early indicators). In the case of SVC, the importance of MMSE reflects SVC’s strength in leveraging clinical assessments in the decision boundaries between the three classes. Moreover, SVC gives more balanced attention to cognitive (MMSE), structural (eTIV), and demographic (EDUC, sex) features. SVC places more weight on clinical scores and broader demographics.

### Sex-based analysis

Evaluating the model’s performance across sexes is critical to ensure its generalizability and fairness in dementia prediction. Without sex-based analysis, potential biases may remain undetected, which reduces the model’s reliability and trustworthiness for clinical applications. For this reason, sex-stratified evaluation was explicitly included in this study as a transparency measure to assess whether aggregate performance masks subgroup-level differences. This is particularly important as the risk factors, incidence, and effects of dementia differ between the sexes, and more evidence is needed on how predictive models can perform differently depending on sex.

To evaluate the sex-specific performance of the best classifiers, a stratified analysis was performed on the test dataset that comprises 30 unique individuals (15 females and 15 males). Despite an equal number of unique participants, the dataset included 35 visits for females and 41 for males, indicating a visit-level imbalance. Classification metrics including precision, recall, F1-score, ROC AUC, PR AUC, and accuracy were calculated separately for male and female participants using both SVC and LGBM models, and the results are given in Table 3.

**Table 3:** Sex-specific performance metrics of the SVC and LGBM classifiers.

Model	Sex	Metrics (%)					
		Precision	Recall	F1-Score	ROC AUC	PR AUC	Accuracy
SVC	Female	69.3	72.5	66.9	83.1	67.3	71.4
	Male	61.8	58.8	54.4	89.9	84.9	58.5
LGBM	Female	68.1	78.3	71.2	82.2	70.4	77.1
	Male	56.8	61.7	57.6	81.7	63.8	63.4

The fairness analysis of SVC and LGBM models applied to the OASIS-2 dataset uncovered notable performance disparities between male and female participants. This imbalance, favoring females across key classification metrics, should be interpreted with caution given the limited sample size and the imbalance in visit counts, which may amplify variability in subgroup-level performance estimates. Rather than conclusively indicating intrinsic sex-related bias in the proposed models, these findings likely reflect a combination of dataset characteristics, sampling variability, and model sensitivity to demographic distributions. Nonetheless, the presence of such disparities highlights important fairness and generalizability considerations that warrant explicit reporting.

The observed disparities are particularly concerning in a medical context, where equitable performance is essential to ensure unbiased diagnoses for all patients. These findings highlight the critical need for targeted interventions to address fairness and enhance model robustness in future studies. Critically, the majority of prior ML studies utilizing the OASIS-2 dataset have not reported sex-stratified performance metrics, relying instead solely on aggregate evaluation scores. This widespread omission risks obscuring systemic biases, as a model may achieve high overall accuracy while failing on subgroups with specific demographics. By explicitly conducting and reporting this stratified analysis, this work prioritizes methodological transparency to uncover latent disparities often hidden within global performance summaries. Consequently, these results establish a more rigorous benchmark for dementia classification and underscore the necessity of subgroup-aware evaluation in the development of clinically equitable ML tools*.*

To mitigate these disparities, future work must move beyond standard performance optimization to prioritize equity. Future research should prioritize applying fairness-aware algorithms, such as those enforcing demographic parity or equal opportunity, that could constrain models to achieve equitable performance across biological sexes. Given the limited sample size of the OASIS-2 dataset, future studies should consider integrating larger, more diverse datasets or leveraging transfer learning to enhance generalizability. Finally, tuning decision thresholds to prioritize recall, especially for males, may minimize missed diagnoses.

## Conclusions

The central contribution of this work is the establishment of a leakage-free and methodologically rigorous benchmark for dementia classification using the OASIS-2 dataset, rather than the development of high-performing predictive models. This study addressed methodological limitations observed in prior OASIS-2 dementia classification research by introducing three key advancements: removing CDR from ML modeling, employing a group-aware data-splitting strategy and developing a suite of engineered temporal features capturing longitudinal changes in clinical and neuroimaging data. By excluding CDR from input features, the target leakage was eliminated, ensuring model performance reflects true predictive signals from demographic and neuroimaging data and enhancing model reliability. The group-aware approach prevented subject-specific information leakage by ensuring that all observations from the same subject were confined to either the training or test set, thus enhancing the reliability of performance estimates. Furthermore, unlike static feature selection or dimensionality reduction methods commonly used, the presented engineered features reflect intra-subject temporal dynamics, such as the rate of cognitive decline or cumulative changes in brain volume, offering richer insights into dementia progression. These contributions establish a more realistic benchmark for assessing classification performance in longitudinal dementia studies.

The efficacy of combining engineered temporal features with the original OASIS-2 dataset features for dementia classification was evaluated, using multiple ML models, including boosting (CB, LGBM), bagging (ET, RF), and SVC. Including engineered features yielded mixed results, with SVC and LGBM benefiting most and emerging as the top-performing models. Although these features did not uniformly improve predictive performance across all models, their evaluation under leakage-free conditions provides important insight into the limitations of current modeling approaches and underscores the challenges of learning reliable temporal signals from sparsely sampled longitudinal data. Combined with the group-aware data-splitting strategy, this innovative feature set can contribute to the development of more generalizable and accurate models for predicting disease outcomes using longitudinal data.

Class-specific performance analysis revealed nuanced differences. For the converted class, SVC outperformed LGBM, correctly classifying 7/12 cases compared to 5/12, indicating better detection of this rare group. Conversely, LGBM perfectly classified all 17 dementia cases, surpassing SVC’s 15/17. This makes it highly effective for identifying individuals with dementia. These findings highlight the importance of model selection based on clinical goals, prioritizing either early detection (higher recall) or minimizing false positives (higher precision). Furthermore, these results simultaneously reveal that current ML models, even with the inclusion of the engineered temporal features in the input set, struggle to robustly identify individuals who convert to dementia. Accordingly, it is essential that future ML-based longitudinal dementia studies report class-specific performance metrics and exercise caution when interpreting high aggregate scores, which may overstate perceived clinical utility. Rather than solely optimizing models for overall performance, future work should explicitly prioritize the reliable identification of the converted group, which represents the most clinically relevant and challenging prediction task.

The SHAP analysis revealed that for both the SVC and LGBM models with the combined features (original and engineered), EDUC and eTIV ranked among the top three most pivotal predictors; however, their importance varied by model architecture. In LGBM, EDUC was the dominant feature, followed by age and eTIV, which reflects a reliance on demographic and structural markers. In SVC, MMSE was the most influential, followed by eTIV and EDUC/sex. This underscores the kernel-based model emphasis on clinical assessments. These differences highlight how model architecture influences feature prioritization in dementia prediction.

The OASIS-2 dataset’s limited sample size and restricted feature range were key constraints of the research that may impact the generalizability and robustness of the findings. To address these limitations, future studies could validate the current findings by incorporating larger and more diverse datasets, such as ADNI or AIBL. Additionally, investigating complex feature interactions and exploring non-linear relationships between clinical and imaging measures could provide deeper insights into the underlying patterns and improve model performance. Such efforts may involve advanced analytical techniques, such as ML algorithms designed to capture non-linear dynamics or feature selection methods to identify critical predictors, thereby strengthening the validity and applicability of the results.

A sex-based analysis revealed performance disparities for both the SVC and LGBM models, with better outcomes for females. This might be due to potential biases that require the development of fairness-aware algorithms to ensure equitable classification across sexes. Furthermore, automated pipelines that dynamically update classification models as patients return for new imaging sessions could be valuable. This approach would ensure that predictions remain aligned with ongoing changes in patients’ cognitive trajectories. Such work could further refine dementia classification models and ultimately enhance early detection and intervention strategies.

## Supporting information

S1 Appendix Trend Analysis of CDR. (PDF)

S2 Appendix The impact of adding the CDR to the feature set. (PDF)
