# Supplementary material for: Explainable machine learning for predicting longitudinal dementia status: Establishing a leakage-free benchmark
Source: PLOS Digit Health. 2026 May 18;5(5):e0001409. doi: 10.1371/journal.pdig.0001409 (PMC13183230; doi:10.1371/journal.pdig.0001409)
Supplement: S1 Appendix — (PDF) [file pdig.0001409.s001.pdf]

S1 Appendix: Trend Analysis of CDR

In the CDR column, a CDR score of 0 indicates the absence of dementia, while scores of 0.5, 1, 2, and 3 correspond to very mild, mild, moderate, and severe dementia, respectively. The primary objective of this section is to investigate the relationship between key neuroimaging, demographic, and clinical variables and their changes as subjects transition from CDR = 0 to CDR > 0. Additionally, it examines progression from one level of dementia to a more severe stage (i.e., increasing CDR scores between visits). Specifically, this analysis focuses on understanding how factors such as age, MMSE scores, nWBV, eTIV, and ASF contribute to the progression of dementia. Due to the lack of variability in the values of EDUC and SES for each subject between visits, these features were excluded from the analysis to maintain the integrity of the dataset. A preliminary screening identified 19 missing values in the SES column and 3 in the MMSE column. The missing values for SES and MMSE were imputed using the median approach. Median imputation was used due to its robustness to outliers and suitability for integer-valued data [38], ensuring that the central tendency of the data is preserved without introducing bias. Following the imputation of missing values, the next step involved normalizing the selected features to a [0,1] range. Normalization is crucial in this context, as it prevents any single feature from disproportionately influencing the results simply due to scale. The prepared data was subsequently analyzed to identify potential inconsistencies within the OASIS-2 dataset. This analysis revealed some unusual patterns in the CDR scores for specific subjects, as detailed in **S1 Table A**.

S1 Table A. Potential inconsistencies in CDR values in the OASIS-2 dataset.

Subject ID	Group	Visit	CDR	MMSE	MR Delay
OAS2_0131	Converted	1	0.5	30	0
		2	0	25	679
OAS2_0064	Demented	1	1	23	0
		2	0.5	26	830
		3	0.5	18	1282
OAS2_0066	Demented	1	1	30	0
		2	0.5	30	497
OAS2_0005	Nondemented	1	0	28	0
		2	0.5	29	1010
		3	0	30	1603
OAS2_0017	Nondemented	1	0	29	0
		3	0.5	27	617
		4	0	30	1861
		5	0	27	2400

Notably, the subject OAS2_0131 was categorized as converted, despite a reduction in the CDR score from 0.5 in the first visit to 0 in the second visit. Similarly, subjects OAS2_0064 and OAS2_0066, both classified within the demented group, exhibited a decrease in CDR scores from 1 to 0.5 across their respective visits. Such changes in CDR scores among individuals in the demented group highlight the complexity of dementia progression and the potential for

fluctuations in clinical evaluations. Conversely, in the nondemented category, subjects OAS2_0005 and OAS2_0017 demonstrated an increase in CDR scores from 0 to 0.5 during their second and third visits, respectively, before reverting back to 0 in subsequent assessments. This pattern of temporary increases in CDR among nondemented individuals could suggest a variety of factors, such as transient cognitive challenges or variations in testing conditions that may influence the scoring. Additionally, some cognitively normal individuals may perform poorly on these assessments due to non-pathological factors such as test anxiety, fatigue, lack of familiarity with the testing format, or temporary distractions, which could contribute to unexpected fluctuations in their CDR scores.

By tracking subjects who transition from a CDR = 0 to a CDR > 0, as well as those who progress from one level of dementia to a more severe stage, as demonstrated by increasing CDR scores between visits, the values of age, EDUC, SES, MMSE, eTIV, nWBV, and ASF were examined both before and after these transitions. The results of this analysis are presented in **S1 Table B**. This table highlights the changes in these key variables as subjects move along the dementia spectrum, providing insights into the relationships between cognitive status and the factors influencing it. By examining these variables, the potential predictors of dementia progression and the nuances of individual experiences in cognitive decline can be assessed. The analysis aims to contribute to a better understanding of the dynamics involved in the transition from nondemented to demented states, ultimately informing clinical assessments and interventions.

S1 Table B. Overview of age, EDUC, SES, MMSE, eTIV, nWBV, and ASF values (normalized) before and after cognitive transitions among subjects in the OASIS-2 dataset.

Group	Subject ID	Visit	CDR	Age	EDUC	SES	MMSE	eTIV	nWBV	ASF
Converted	OAS2_0018	3	0	0.74	0.47	0	0.96	0.33	0.36	0.53
		4	0.5	0.84	0.47	0	0.88	0.35	0.27	0.50
	OAS2_0020	1	0	0.53	0.82	0	0.96	0.53	0.25	0.32
		2	0.5	0.58	0.82	0	0.92	0.56	0.17	0.31
	OAS2_0031	2	0	0.74	0.35	0.50	1	0.38	0.39	0.48
		3	0.5	0.82	0.35	0.50	0.92	0.40	0.27	0.46
	OAS2_0041	2	0	0.34	0.59	0	0.92	0.21	0.64	0.67
		3	0.5	0.39	0.59	0	0.92	0.23	0.60	0.65
	OAS2_0054	1	0	0.66	0.71	0	0.96	0.18	0.29	0.72
		2	0.5	0.71	0.71	0	0.77	0.19	0.20	0.70
	OAS2_0092	1	0	0.61	0.35	0.25	0.92	0.31	0.54	0.55
		2	0.5	0.63	0.35	0.25	0.88	0.32	0.43	0.54
	OAS2_0103	1	0	0.24	0.59	0	1	0.33	0.55	0.53
		2	0.5	0.37	0.59	0	1	0.35	0.40	0.50
	OAS2_0118	1	0	0.18	0.47	0.75	1	0.45	0.78	0.40
		2	0.5	0.29	0.47	0.75	0.85	0.47	0.75	0.38
	OAS2_0127	1	0	0.50	0.71	0	0.96	0.60	0.44	0.27
		2	0.5	0.55	0.71	0	0.96	0.61	0.39	0.26

	OAS2_0133	1	0	0.47	0.35	0.50	0.96	0.41	0.45	0.44
		3	0.5	0.55	0.35	0.50	0.92	0.43	0.22	0.42
	OAS2_0144	1	0	0.45	0.59	0	1	0.67	0.37	0.22
		2	0.5	0.50	0.59	0	1	0.69	0.33	0.20
	OAS2_0145	1	0	0.21	0.59	0.50	1	0.21	0.80	0.67
		2	0.5	0.34	0.59	0.50	0.96	0.20	0.66	0.69
	OAS2_0176	2	0	0.71	0.59	0.25	1	0.33	0.27	0.53
		3	0.5	0.76	0.59	0.25	1	0.34	0.18	0.52
**Demented**	OAS2_0007	1	0.5	0.29	0.59	0.25	0.92	0.28	0.54	0.59
		3	1	0.34	0.59	0.25	0.88	0.29	0.43	0.58
	OAS2_0014	1	0.5	0.42	0.59	0.50	0.65	0.55	0.27	0.31
		2	1	0.45	0.59	0.50	0.46	0.54	0.27	0.32
	OAS2_0028	1	0.5	0.11	0.71	0.25	0.69	0.49	0.48	0.36
		2	1	0.16	0.71	0.25	0.65	0.51	0.38	0.35
	OAS2_0046	1	0.5	0.61	0.53	0.25	0.62	0.41	0.55	0.44
		2	1	0.66	0.53	0.25	0.69	0.42	0.54	0.43
	OAS2_0050	1	0.5	0.29	0.35	0.75	0.62	0.39	0.41	0.46
		2	1	0.32	0.35	0.75	0.50	0.42	0.26	0.43
	OAS2_0079	1	0.5	0.24	0.35	0.75	0.73	0.38	0.60	0.47
		2	1	0.29	0.35	0.75	0.46	0.43	0.42	0.42
	OAS2_0087	1	1	0.95	0.65	0	0.85	0.40	0.20	0.45
		2	2	1	0.65	0	0.65	0.44	0.08	0.41
	OAS2_0089	1	0.5	0.26	0.35	0.25	0.96	0.36	0.25	0.49
		3	1	0.32	0.35	0.25	0.88	0.36	0.21	0.49
	OAS2_0104	1	0.5	0.26	0.59	0	0.81	0.51	0.27	0.34
		2	1	0.29	0.59	0	0.50	0.51	0.21	0.35
	OAS2_0114	1	0.5	0.42	0.35	0.25	0.88	0.23	0.43	0.64
		2	1	0.47	0.35	0.25	0.88	0.23	0.33	0.65
	OAS2_0120	1	1	0.42	0.47	0.50	0.81	0.34	0.36	0.52
		2	2	0.47	0.47	0.50	0.42	0.33	0.29	0.53
	OAS2_0150	1	0.5	0.34	0.35	0.50	1	0.26	0.39	0.61
		2	1	0.39	0.35	0.50	0.88	0.28	0.36	0.59
	OAS2_0160	1	0.5	0.42	0.35	0.25	0.88	0.50	0.32	0.35
		2	1	0.47	0.35	0.25	0.96	0.52	0.31	0.34
	OAS2_0164	1	1	0.45	0.82	0	0.73	0.68	0.58	0.21
		2	2	0.50	0.82	0	0.81	0.67	0.60	0.21
	OAS2_0181	1	0.5	0.37	0.35	0.25	0.85	0.07	0.46	0.88
		2	1	0.39	0.35	0.25	0.90	0.07	0.51	0.88
	OAS2_0184	1	0.5	0.32	0.59	0.50	0.77	0.28	0.46	0.59

2	1	0.34	0.59	0.50	0.65	0.27	0.33	0.60

S1 Fig A depicts the percentage differences in normalized values of MMSE, Age, eTIV, nWBV, and ASF for OASIS-2 subjects who exhibited an increase in CDR values. Each subplot represents a different variable, allowing for a comparative analysis of how these metrics change among individuals. The percentage differences are plotted against the subject IDs, with each dot representing a subject's change over time, depicted in a light blue color for clarity. A red dashed line at y=0 serves as a baseline, indicating no change, while orange lines representing the 10th and 90th percentiles provide additional context about the variability within the data.

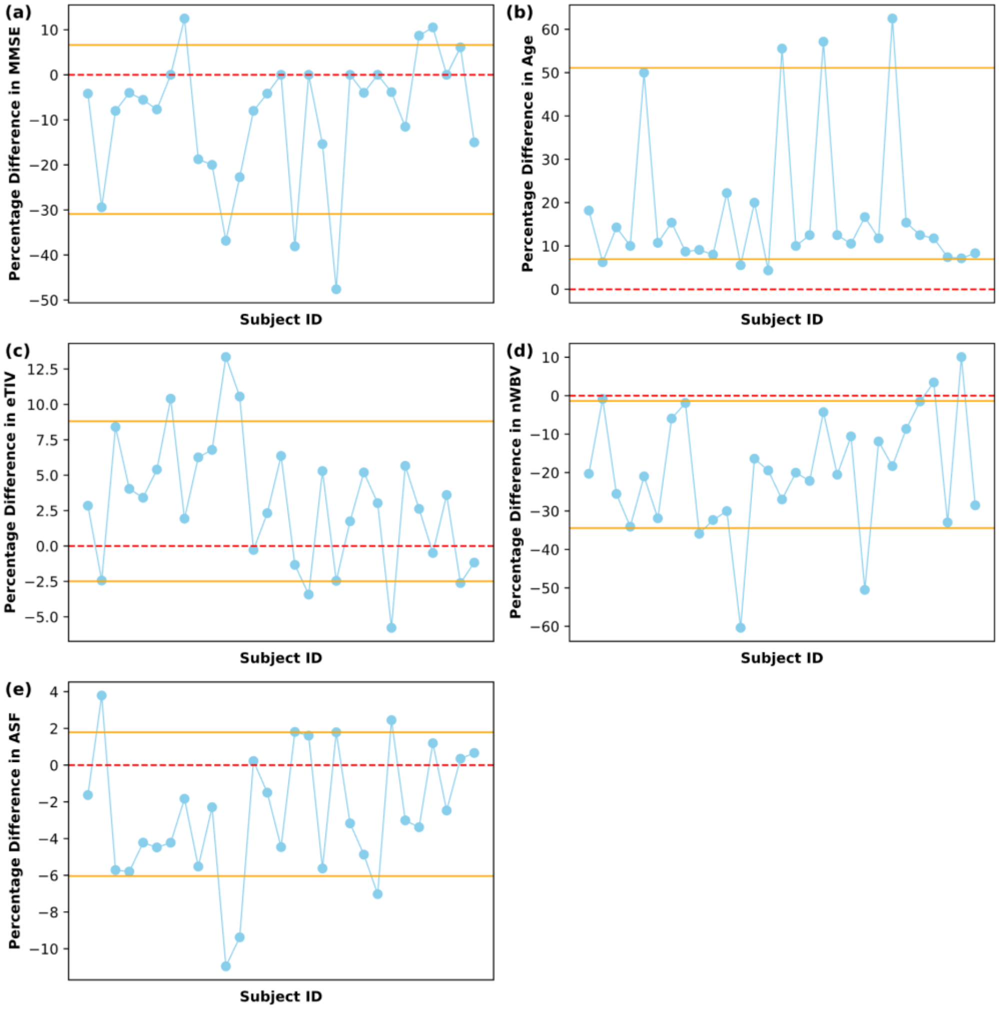

S1 Fig A. Percentage differences in normalized MMSE, Age, eTIV, nWBV, and ASF for OASIS-2 subjects with increasing CDR values. Each line represents an individual subject, with a red dashed line at y = 0 indicating no change and orange lines marking the 10th and 90th percentiles.

S1 Fig B consolidates all features into a single plot to provide a more integrated visualization of how these features change simultaneously. This approach allows for a clearer comparison of trends

across features, making it easier to observe overall patterns and potential correlations among the variables.

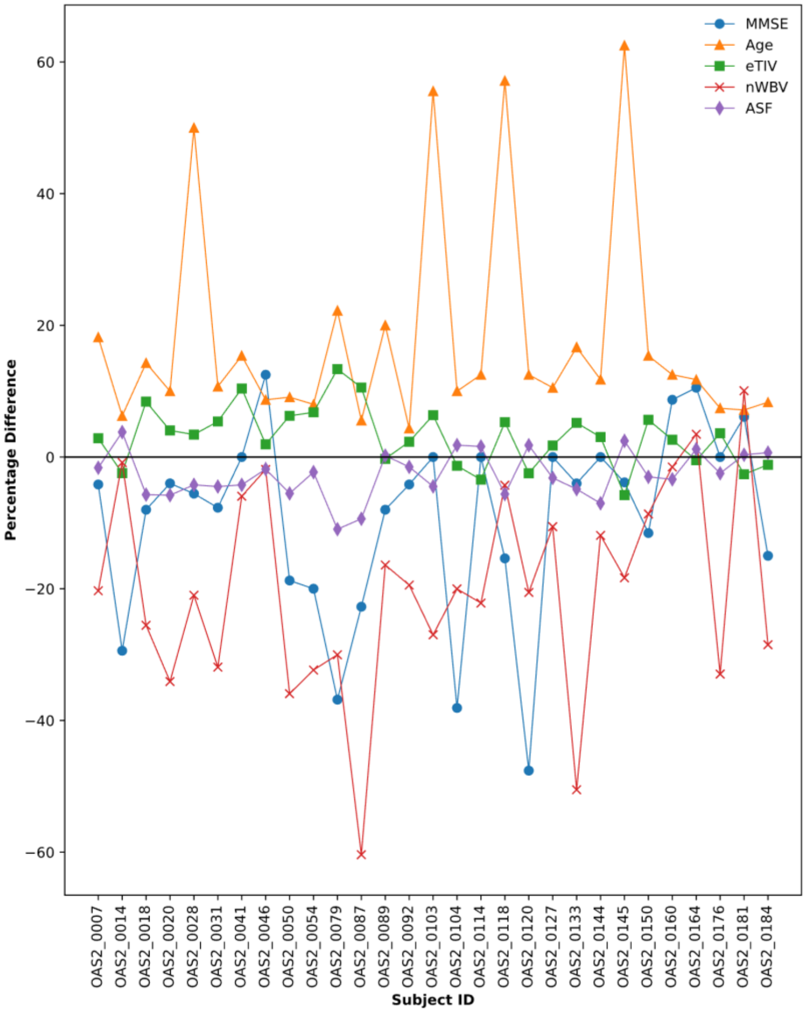

S1 Fig B. Combined visualization of percentage differences in normalized MMSE, Age, eTIV, nWBV, and ASF for OASIS-2 subjects with increasing CDR values.
