# Supplementary material for: Explainable machine learning for predicting longitudinal dementia status: Establishing a leakage-free benchmark
Source: PLOS Digit Health. 2026 May 18;5(5):e0001409. doi: 10.1371/journal.pdig.0001409 (PMC13183230; doi:10.1371/journal.pdig.0001409)
Supplement: S2 Appendix — (PDF) [file pdig.0001409.s002.pdf]

S2 Appendix: The impact of adding the CDR to the feature set

As can be seen in **S2 Table A**, when CDR is introduced as an input variable, every algorithm exhibits a noticeable change in classification performance. The largest improvements are observed for LGBM and SVC. However, tree-based ensembles respond heterogeneously. Furthermore, for all models, the inclusion of engineered features along with the original features consistently resulted in enhanced performance across all metrics.

S2 Table A. Test-set performance of the ML classifiers on OASIS-2 when CDR is included in the predictor sets

Model	Input	Metrics (%)					
		Precision	Recall	F1-Score	ROC AUC	PR AUC	Accuracy
CB	Original	77	72	74	84	74	72
	Combined*	85	85	85	95	90	85
ET	Original	79	41	35	88	70	41
	Combined	83	57	57	93	83	57
RF	Original	62	61	59	83	65	61
	Combined	83	83	83	91	80	83
LGBM	Original	80	82	80	91	83	82
	Combined	92	92	92	93	90	92
SVC	Original	86	86	86	92	87	86
	Combined	94	94	93	98	96	94

**Both original and engineered features*

These outcomes confirm that CDR is a highly informative covariate; however, because the class labels in OASIS-2 are themselves derived from CDR, its inclusion introduces target leakage. For clinically realistic models, the leakage-free results in the main manuscript remain the appropriate reference.
